# Bilateral Traumatic Fracture of Neck of Femur in a Child: A Case Report

**DOI:** 10.5704/MOJ.1307.001

**Published:** 2013-07

**Authors:** D Dhar

**Affiliations:** Department of Orthopaedics, Regional Referral Hospital, Nizwa, Oman

## Abstract

Bilateral femoral neck fractures are rare in the pediatric age group, and only a few cases have been reported following major trauma in children. A 9-year old girl presented with bilateral femoral neck fractures following a motor vehicle accident. The patient was managed with early operative fixation of the fractures with a successful outcome. This case highlights the importance of awareness of the occurrence of bilateral femoral neck fractures in the polytrauma patients. This case is presented due to its rarity.

## Introduction

Fractures of the femoral neck are rare in children. There is however, a high incidence of complications from these injuries, such as avascular necrosis of the femoral head and non–union 1. Bilateral femoral neck fractures have been reported in adults following trauma and underlying bone disease. There have been few reports on traumatic bilateral femoral neck fractures in the pediatric age group. In this case report of this rare injury, we highlight the need for early diagnosis and surgical intervention so as to achieve a successful outcome.

## Case Report

**Fig. 1:**
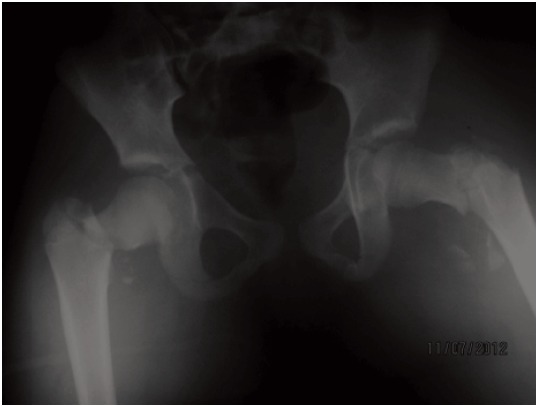
: Anterioposterior radiograph of pelvis showing bilateral displaced fracture neck femur.

**Fig. 2:**
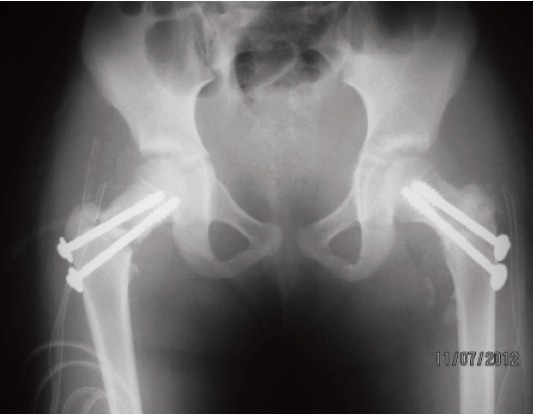
: Anterioposterior radiograph of pelvis after internal fixation of femoral neck fractures with cancellous screws.

**Fig. 3:**
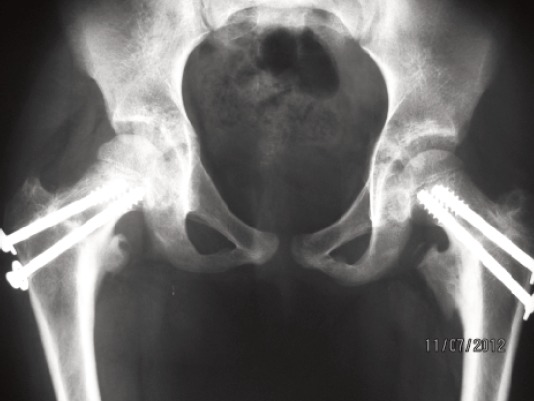
: Anterioposterior radiograph of pelvis showing both fracture neck femur healed with implants in situ.

**Fig. 4:**
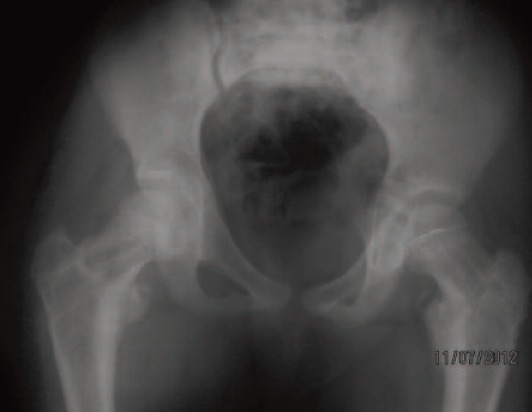
: Anterioposterior radiograph of pelvis immediately after cancellous screw removal.

A 9-year old girl presented to the Accident and Emergency department with bilateral closed femoral neck fractures sustained in a motor vehicle accident. The patient had associated head injury, a closed comminuted fracture of the proximal right tibia, and fracture of the right pubic ramus. On arrival she was hypotensive, drowsy and irritable, and had a GCS of 8/15. The right lower limb was externally rotated and there was a deformity of the right leg. Attempted movement around the hips was painful. She was resuscitated and intubated endotracheally at the Accident and Emergency department. Both lower limbs required splinting. Radiographs of the pelvis showed a displaced right trans-cervical (type II) and a left cervico–trochanteric (type III) femoral neck fracture (Fig 1) as per the Delbert classification. CT scan of the brain showed intracerebral hemorrhage. The patient was admitted to the pediatric intensive care unit and was jointly managed by the general surgeon, neurosurgeon and the orthopedic team. Above knee skin traction were applied for both lower limb. Forty hours later, she underwent open reduction and fixation of both femoral neck fractures, with two 4.0 mm partially threaded cancellous screws avoiding the femoral growth plates. The post-operative period was uneventful and on the 10th post-operative day, a bilateral hip spica cast incorporating the right tibia fracture was applied. The right tibia fracture was managed conservatively. The hip spica was removed at 9 weeks when good healing of the fractures at the hips and right tibia was observed (Fig 2). Physiotherapy was started for mobilization of the joints and this was followed by axillary crutch gait training. A follow-up review showed that the fractures had healed well (Fig 3) by 13 months post injury. The cancellous screws were removed from both hips (Fig 4) at 14 months post injury. At 24 months, the radiographs did not show any sign of avascular necrosis of the femoral heads. Range of movements of both hip joints was full and painless. There was no in limb length discrepancy.

## Discussion

Femoral neck fractures comprise less than 1% of paediatric and adult fractures 2. Bilateral femoral neck fractures are rare and only a few cases have been reported in the literature. These fractures result mainly from high impact trauma as in motor vehicle accidents and falls from a height. Occasionally, they occur secondary to underlying metabolic disorders 2. The mechanism of injury in our case was that of high impact trauma from a motor vehicle accident. Other mechanisms of injury have been described such as an indirect abduction and external rotation force hinging the femoral neck against the acetabular rim causing the fracture^3^.

Avascular necrosis (AVN) remains the most dreaded complication following these fractures, reported to be 18% to 30% by various authors 1. The incidence of AVN has been reported to be 41% when hip decompression was not done compared with 8% in cases treated with early hip decompression^4^. In our case, avascular necrosis has not been observed up until 24 months post injury. The main factors influencing AVN rate are the initial fracture displacement, the degree of initial insult, and the timing of surgical fixation together with hip decompression. Operative fixation should be carried out preferably within 48 hours of fracture. Our case was operated on 40 hours after admission after optimization of her general condition and this is consistent with accepted guidelines. Other complications which can occur are non–union, coxa vara, coxa valga, leg length discrepancy, arthritic changes and premature closure of proximal femoral epiphysis 1. Our patient did not have any of these complications.

In a recent systematic review Yeranosian et al^5^ have reported that operative treatment or the type of reduction do not affects the rate of AVN, nonunion or premature physeal closure. Capsular decompression had no effect on AVN but delay in treatment beyond 24 hours was associated with a higher incidence of AVN.

In summary this is a report of a rare case of bilateral femoral neck fracture in a child. Early diagnosis and surgical intervention can lead to a good outcome. Proper assessment of pelvic radiographs especially in patients involved in high impact trauma is mandatory. A high index of suspicion will lessen the chances of missing such injuries in children.
